# The Effects of Physical Activity and the Consequences of Physical Inactivity in Adult Patients with Congenital Heart Disease During the COVID-19 Pandemic

**DOI:** 10.3390/jfmk9040226

**Published:** 2024-11-08

**Authors:** Elettra Pomiato, Rosalinda Palmieri, Mario Panebianco, Giulia Di Già, Marco Della Porta, Attilio Turchetta, Massimiliano Raponi, Maria Giulia Gagliardi, Marco Alfonso Perrone

**Affiliations:** 1 Bambino Gesù Children’s Hospital IRCCS, 00165 Rome, Italy; elettra.pomiato@opbg.net (E.P.); rosalinda.palmieri@opbg.net (R.P.); mario.panebianco@opbg.net (M.P.); giulia.digia@opbg.net (G.D.G.); marco.dellaporta@opbg.net (M.D.P.); attilio.turchetta@opbg.net (A.T.); massimiliano.raponi@opbg.net (M.R.); mgiulia.gagliardi@opbg.net (M.G.G.); 2 Department of Clinical Sciences and Translational Medicine, University of Rome Tor Vergata, 00133 Rome, Italy

**Keywords:** ACHD, COVID-19, physical activity, lockdown, exercise tolerance

## Abstract

Background: The ongoing COVID-19 pandemic has infected more than 500 million people worldwide. Several measures have been taken to reduce the spread of the virus and the saturation of intensive care units: among them, a lockdown (LD) was declared in Italy on 9 March 2020. As a result, gyms, public parks, sports fields, outdoor play areas, schools, and multiple commercial activities have been closed. The consequences of physical inactivity can be dramatic in adult patients with congenital heart disease (ACHD), in which the benefit of regular exercise is well known. In this study, we investigated the effects of reduced physical activity during the COVID-19 pandemic on ACHD’s exercise capacity. Materials and Methods: Patients who performed exercise or cardiopulmonary exercise tests from October 2019 to February 2020 and one year after lockdown with the same protocol were retrospectively enrolled in our database. Inclusion criteria: ACHD patients aged ≥ 18 years old under regular follow-up. Exclusion criteria: significant clinical and/or therapeutic changes between the two tests; significant illness occurred between the two tests, including COVID-19 infection; interruption of one of the tests for reasons other than muscle exhaustion. Results: Thirty-eight patients (55.6% males) met the inclusion criteria. Before the lockdown, 17 patients (group A) were engaged in regular physical activity (RPA), and 20 patients (group B) had a sedentary lifestyle. After LD, in group A, (a) the weekly amount of physical activity reduced with statistical significance from 115 ± 46 min/week to 91 ± 64 min/week (−21%, *p* = 0.03); (b) the BMI did not change; (c) the duration of exercise test and VO2 max at cardiopulmonary exercise test showed a significant reduction after the LD. In group B, BMI and exercise parameters did not show any difference. Conclusions: The COVID-19 pandemic dramatically changed the habits of ACHD patients, significantly reducing their possibility to exercise. Our data analyzed in this extraordinary situation again demonstrated that physical inactivity in ACHD worsens functional capacity, as highlighted by VO2 max. Regular exercise should be encouraged in ACHD patients to preserve functional capacity.

## 1. Introduction

The COVID-19 pandemic has affected over 500 million individuals globally and significantly changed the global health, economy, and social and behavioral habits worldwide [1]. During the peak and post-peak pandemic phases, different countries have promoted different degrees of social distances and restrictions to mitigate the viral spread and alleviate strain on healthcare systems. The rapid spread of the virus prompted governments and health authorities to implement a range of public health measures aimed at curbing transmission, including varying degrees of social distancing, mandatory quarantines, and restrictions on movement. While crucial to slowing the spread of the virus and easing the burden on health systems, these measures also led to significant and widespread disruptions in daily activities, particularly those related to social interaction and physical activity (PA) [1,2,3]. Italy, among the first severely affected countries, implemented a nationwide lockdown (LD) on 9 March 2020, shuttering various communal spaces like gyms, swimming pools, parks, and schools [2]. These closures were pivotal in curbing virus transmission but have had several unintended repercussions. Although published data are heterogeneous, a decline in all the physical activity measures was noted [2,3,4,5].

Physical exercise is widely accepted as part of a healthy lifestyle [5,6]. A plethora of experimental and clinical studies have conclusively demonstrated that regular physical activity (RPA) is associated with a reduced risk of occurrence and mortality rate from non-communicable diseases [6,7,8]. In particular, much evidence from large prospective population-based observational studies has shown that maintaining or improving physical fitness is associated with a lower risk of cardiovascular disease, diabetes, obesity, chronic obstructive pulmonary disease, and cancer [7,8].

On the other hand, several studies have reported that physical inactivity leads to an increased risk of all-cause mortality, poor general health, and lower life expectancy, with high morbidity and mortality rates in patients with non-communicable diseases [8].

In particular, cardiovascular disease (CVD) is the leading cause of morbidity and mortality worldwide. Among the numerous risk factors that predispose to the development and progression of CVD, a sedentary lifestyle, characterized by persistently low levels of physical activity, is now recognized as a major contributor to poor cardiovascular health. In contrast, regular exercise and physical activity are associated with significant health benefits and a significantly lower risk of CVD. Several long-term studies have shown that increased physical activity is associated with a reduction in hospitalization rates, overall cardiovascular mortality, and increased life expectancy [8].

PA can improve insulin sensitivity, alleviate plasma dyslipidemia, reduce high blood pressure and blood viscosity, promote endothelial nitric oxide production, and improve the metabolic profile to protect the heart and vessels [8].

Over the years, the benefits of PA have been demonstrated in patients with different cardiovascular diseases such as hypertension, ischemic heart disease, heart valve disease, heart failure, and cardiomyopathy [7,8]. More recently, several studies have demonstrated the effectiveness of physical activity in improving physical, psychological, and social conditions, even in patients with congenital heart disease [9,10].

Congenital heart disease (CHD) is defined as a heterogeneous group of pathologies characterized by structural alterations of the heart or large vessels already present during fetal life. It is the most common malformation at birth (about 40%) and affects approximately one newborn in every 100 live births [10,11].

Until a few decades ago, neonatal mortality was extremely high, and only a few patients reached adulthood [9,11]. Today, thanks to clinical and surgical innovations, approximately 80–85% of children born with congenital heart disease manage to survive to adulthood [9,10]. Therefore, if in the past, the ratio of children/adults with congenital heart disease showed that survival to adulthood was low, in recent years, the percentage of adult patients with congenital heart disease has increased significantly. Indeed, it is estimated that in the next few years, the number of adult patients with congenital heart disease in the world will exceed the number of children [9,11,12].

We are, therefore, facing a new and growing population of adult congenital heart patients, previously identified with the acronym GUCH (Grown Up Congenital Heart), while currently ACHD (Adult with Congenital Heart Disease) is preferred. The management of these patients can be challenging and requires an intense multidisciplinary integration that involves figures who operate both in the field of pediatric cardiology and cardiac surgery, as well as in the field of adult cardiology and cardiac surgery [11,12,13].

In fact, this population is decidedly heterogeneous, as some congenital heart diseases can be tolerated with hemodynamic stability for decades and become clinically evident in adult life. Other patients undergo palliative or radical correction of their heart disease in childhood but often require medical and surgical care long after the corrective surgery. Added to this is the need to develop, for this particular type of patient, specific multidisciplinary counseling programs that concern the sexual sphere, pregnancy, work activity, and indeed physical activity and sport [9,11,12,13,14].

Today, it is well-established that physical activity benefits physical and mental health in adult patients with congenital heart disease [6,11,12]. Recent studies have shown that regular exercise increases cardiopulmonary performance, improves cardiac biomarker values, increases exercise tolerance, and improves the quality of life in adult patients with congenital heart disease, including complex ones [9,11,12,13,14]. However, in the past, the evidence of such benefits in patients with congenital heart disease has been historically weak, and the most complex congenital heart disease has traditionally been cautioned against moderate or vigorous PA due to safety concerns [13,14,15]. However, over the last 25 years, we assisted in a paradigm shift [7], and nowadays, the majority of the literature favors physical activity in adult patients with congenital heart disease (ACHD) following the appropriate screening with few exceptions [14,15,16,17].

In this population, regular exercise has been shown to be beneficial in the short and long term [8,9] and has become paramount in the management and treatment of cardiac conditions and in sustaining overall health [10,11,12,13,14,15,16]. However, during LD, the closure of exercise facilities and outdoor areas significantly disrupted their ability to engage in physical activity. An abrupt halt in exercise routines can precipitate various adverse outcomes, exacerbating existing cardiac issues and impeding recovery and rehabilitation efforts. Cardiovascular deconditioning, muscle weakness, weight gain, and psychological distress are among the potential consequences of prolonged physical inactivity in this population. This study aimed to investigate the effects of reduced PA and LD during the COVID-19 pandemic on the cardiopulmonary capacity of ACHD.

## 2. Materials and Methods

Adult patients with congenital heart disease who had been followed for continuity of care since childhood at our institution were retrospectively examined. Patients ≥ 18 years old who completed an exercise test (ETT) or a cardiopulmonary exercise test (CPET) between October 2019 and February 2020 (i.e., before COVID-19 LD) and repeated the same test one year after were selected for the analysis. Patients with significant changes in their cardiac condition (i.e., worsening in NYHA class, need for medication up-titration, hospital admission, percutaneous, or surgical interventions) between the tests, patients with severe non-cardiac illness occurred between the two tests (including COVID-19 infection), and patients who requested interruption of one of the tests for reasons other than muscle exhaustion were excluded from the analysis. Age, sex, type of congenital heart disease, NYHA class, and level of physical activity were recorded as baseline parameters. All information was collected from the patient’s medical records. In our institution, regular physical activity (minutes per week) is regularly asked in detail during the visit and before the exercise test in ACHD patients. This information is routinely collected during the clinical evaluation both for clinical purposes and to set the appropriate exercise protocol when the exercise test is requested.

Patients were divided according to their level of physical activity before the LD in group A (regular physical activity) and group B (sedentary patients).

The following parameters were investigated before the lockdown and one year after: body mass index (BMI), duration of exercise test, and maximal oxygen consumption (VO2 max), where available.

### 2.1. Exercise Test and Cardiopulmonary Exercise Test

ETT was performed on a treadmill (Technogym, Cesena, Italy) following Bruce protocol [9]. Electrocardiogram (ECG) and oxygen saturation (SpO_2_) were monitored continuously. Blood pressure was monitored at rest, at each Bruce stage, at maximal effort, and during the recovery at 30 s, 60 s, and every two minutes after.

When clinically required, CPET was on a treadmill (Technogym, Cesena, Italy) following Bruce protocol [9]. ECG and SpO_2_ were monitored continuously. Blood pressure was monitored at rest, at each Bruce stage, at maximal effort, and during the recovery at 30 s, 60 s, and every two minutes after. Ergospirometry with breath-by-breath sampling was used to assess a full set of CPET parameters. Gas exchange and CPET parameters were processed by a COSMED gas analyzer (COSMED, Rome, Italy). For our study, we decided to focus on the VO2 max.

Regardless of the test modality, all the patients were encouraged to exercise until exhaustion and asked to specify the reason for the test interruption.

This study was approved by the ethics committee of Bambino Gesù Children’s Hospital IRCCS (protocol code 2610), and all patients signed informed consent. It was conducted in accordance with the Declaration of Helsinki.

### 2.2. Statistical Analysis

Continuous variables were presented as means ± SD if they were normally distributed or with median and range in case of skewed distribution. The Shapiro–Wilk normality test was used to test the characteristics of the distribution. Categorical variables were presented as counts and percentages. 

Differences in the rate of physical activity, weight, BMI, duration of exercise, maximal HR, and VO2 max pre- and post-lockdown in the same Group were assessed by paired Student t-test or paired nonparametric tests (Wilcoxon test), as appropriate.

Independent sample *t*-test was used to evaluate differences in VO2 max between group A and group B.

Additionally, repeated measures ANOVA was conducted to examine the effect of time and physical exercise on VO2 max. The results of the analysis are expressed as F-factor, *p*-value, and partial eta squared. The normality assumption was verified using Shapiro–Wilk tests, which were insignificant for any group level. The sphericity assumption was assumed as met as VO2 max had only two levels. Levene’s test was used to test the homogeneity of variances. Post-hoc tests for time * group analysis were performed using Bonferroni correction.

A *p*-value < 0.05 was considered statistically significant. All analyses were performed using SPSS (version 20.0).

## 3. Results

Between October 2019 and February 2020, 162 ACHD patients completed a functional evaluation at our institution. Among them, 37 (55.6% males, aged 26 ± 6 years) met the inclusion criteria. VO2 max was available in 29 (78.3%). The baseline characteristics of the patients are summarized in Table 1.

According to the level of physical activity before the lockdown, 17 patients (46%) were included in group A (regular physical activity) and 20 patients (54%) in group B (sedentary). Patients in group A practiced monitored physical activity at least two sessions per week, 30 min of walking or running on a treadmill or exercise bike, with an intensity of not less than 50–60% of maximum heart rate (HR). Characteristics of the exercise of the two groups at baseline (pre-LD) and after one year are summarized in Table 2.

During the COVID-19 pandemic, four patients of group A (24%) completely stopped their training, while others reduced it. Overall, the amount of regular physical activity (RPA) in group A reduced significantly from 115 ± 46 min/week to 91 ± 64 min/week (−21%, *p* = 0.03).

Furthermore, in the patients of group A, the duration of exercise and VO2 max reduced significantly after the lockdown (*p* = 0.009 and *p* = 0.015, respectively). The maximal achieved HR, weight, and BMI did not show statistically significant changes before and after the lockdown.

In group B, there were no significant differences in the analyzed parameters.

**Figure 1:**
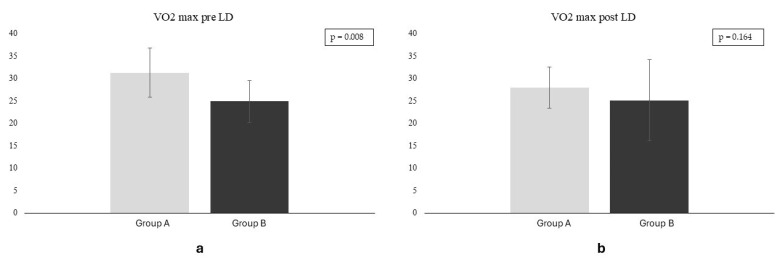
VO2 max in groups A and B before (**a**) and after (**b**) the lockdown. VO2 max is displayed in mL/kg/min. LD: lockdown.

Before LD, the VO2 max was significantly higher in group A compared to group B (*p* = 0.008), but the difference was not statistically significant post-LD (*p* = 0.164) (Figure 1).

A repeated measures ANOVA was conducted to examine the effect of time and physical exercise (groups A and B) on VO2 max. The main effect of time was significant, with F-value = 6.31, *p* < 0.022, and partial eta-squared = 0.271. There was also a significant interaction between time and group (time × group), with F-value = 4.85, *p* < 0.042, and partial eta-squared = 0.222. There was a significant interaction effect between VO2 max and group, with F-value = 9.89, *p* < 0.006, and partial eta-squared = 0.368. Post-hoc tests for the time x group revealed that VO2 pre-LD was significantly higher in group A compared to group B (mean difference 8.098 mL/kg/min, *p* Bonferroni 0.011). Also, in group A, the VO2 pre-LD was significantly higher than the VO2 post-LD (mean difference 3.600 mL/kg/min, *p* Bonferroni 0.039). There was no difference in the VO2 max pos- LD between group A and group B (mean difference 4.734 mL/kg/min, *p* Bonferroni 0.261), nor in VO2 max post-LD of group A and the VO2 max pre-LD if group B (mean difference 4.498 mL/kg/min, *p* Bonferroni 0.327).

## 4. Discussion

The COVID-19 pandemic profoundly changed the habits and lifestyles of patients worldwide. In particular, during the early phase of the disease, drastic measures such as lockdowns were necessary to limit the spread of the virus. Our study investigated retrospectively how the COVID-19 pandemic influenced PA and exercise tolerance in two cohorts of adult patients with congenital heart disease.

### 4.1. Physical Activity

Although the majority of the published studies seem to support an overall reduction in the PA [2,17], the results of a recent meta-analysis highlighted a degree of heterogeneity [5]. The authors described a total sample size of 119,094 participants from 14 countries worldwide, with participants aged between four and ninety-three years [5]. Thirty-two studies revealed a significant decline in PA, whereas only five studies found a significant increase in PA during the COVID-19 pandemic. Instead, fourteen studies revealed mixed results. The data are sometimes in contrast, especially due to the age of the subjects studied [5]. For example, the trend toward a decrease in PA levels in children and adolescents has not always been significant since the decrease in sports and indoor PA was counterbalanced in some studies by an increase in active daily and outdoor activities [18,19], while in adults, some studies suggested an increase in walking during the pandemic [20]. However, the majority of the studies suggest a significant decrease in the daily PA in the cohort 20–35 years of age [21,22]. Furthermore, another study conducted on children and adolescents with congenital heart disease showed that the percentage of patients engaged in regular physical activity decreased significantly during the pandemic, while only a small percentage of sedentary patients started to practice physical activity during the LD [17].

The reduction of the weekly amount of PA in our cohort reached 21% (*p* = 0.03) and reflected the impact that isolation measures had on ACHD. Such a difference could be explained both by the strict policy endorsed by the Italian Government during the initial phase of the pandemic and the recommendation endorsed by the European Society of Cardiology, promoting isolation homeworking and homeschooling in patients with complex congenital heart disease [23,24].

Interestingly, in contrast with other studies [25,26], the reduction in PA was not associated with an increase in weight or BMI. Instead, the authors of a previous study showed a significant increase in BMI in pediatric patients with congenital heart disease who had decreased physical activity during LD [17]. In our cohort, weight did not increase significantly. This could be attributed to the fact that adult patients with congenital heart disease, being more aware of their condition, were, to some extent, more attentive to their eating habits during the lockdown compared to the general population.

### 4.2. Exercise Tolerance

The duration of exercise remained stable in the sedentary patients (median 630 vs. 578 s, *p* = 0.118) while decreased significantly in the patients of group A (625 vs. 581 s, *p* = 0.009). Similarly, the VO2 max remained stable in the sedentary cohort (24.9 ± 4.7 mL/kg/min pre-LD vs. 25.1 ± 5.5 mL/kg/min post-LD, *p* = 0.820) but reduced significantly after the LD in the group of patients who practiced physical exercise (from 31.3 ± 5.5 mL/kg/min pre-LD vs. 28.0 ± 4.1 mL/kg/min post-LD, *p* = 0.015). Furthermore, of great relevance is that before the COVID-19 pandemic the VO2 max was significantly higher in the active cohort (group A) than in sedentary subjects (group B) (−6.4 mL/kg/min corresponding to −20.4% in the sedentary cohort, *p* = 0.008), but the difference in exercise performance between the two groups halved after the LD (VO2 max −2.9mL/kg/min corresponding to −10%) and is not statistically significant (*p* = 0.164).

Both exercise duration and VO2 max are critical measures for assessing the functional capacity in patients and healthy subjects. Exercise duration provides a practical measure of endurance and daily functional ability, while VO2 max offers a detailed assessment of aerobic capacity and cardiorespiratory fitness. 

Together, these parameters are pivotal in evaluating disease severity, monitoring progression, and guiding treatment strategies. In particular, in ACHD patients, VO2 max at CPET is related to the NYHA functional class and is a predictor of morbidity at mid-term follow-up [10,11,12,13,14,15,16,18,27,28,29,30,31,32]. The data from our study are comparable with those from another previous study where children and adolescents with congenital heart disease were studied, comparing the exercise test before and after LD [17]. In this study, the percentage of patients engaged in RPA decreased significantly during the pandemic, and BMI increased significantly, except among the subjects who began RPA during the lockdown, whereas test duration did not decrease significantly overall but increased in this last subgroup [17].

Exercise capacity generally declines with age [26,27,28]. This reduction is attributed to several physiological changes that occur in the aging process. The decline in maximum heart rate, which decreases at a rate of approximately 0.7 beats per year, together with lower ventilatory efficiency and reduced oxygen exchange during exercise and age-related decreases in muscle mass and strength, can contribute to reduced exercise tolerance [29,30,31,32].

In individuals with congenital heart disease, these age-related declines in exercise capacity can be more pronounced [33,34]. The presence of residual hemodynamic abnormalities, prior surgeries, and the chronic burden of the disease further complicate the natural decline in exercise function [32,33,34].

In our study, a decrease in exercise capacity was noted in group A (duration −44 s and VO2 max −3.3 mL/kg/min) but not in group B. This is unlikely to be explained solely by age-related exercise intolerance as the two exercise tests were performed within a one-year time frame, and the reduction in cardiopulmonary fitness was not observed in patients who were already sedentary.

Moreover, the exercise protocol and equipment did not change between the two tests. It is well known that a repeated exercise test, administered after a brief interval, typically results in improved performance generally attributed to increased familiarity with the test protocols and the testing environment, and this could have contributed to limiting the drop in functional capacity, as shown in our sedentary cohort.

This aspect, combined with the observed reductions in both exercise duration (−7%) and VO2 max (−10.5%), supports the hypothesis that inactivity plays a significant role beyond the natural, time-related decline in exercise capacity.

In conclusion, our analysis suggests that a reduction of PA in adults with congenital heart disease during the LD led to a significant worsening of functional capacity.

Exercise is considered part of the active treatment of almost all congenital cardiac conditions, being contraindicated in only a few exceptions [8,35,36]. Previous studies have shown its pivotal role in improving functional capacity, symptoms, and cardiac biomarkers in both simple and complex ACHD [9,16,17], as well as general health, exercise tolerance, and quality of life in adults with congenital heart disease being exercise training is a high-benefit, low-risk intervention in most patients with congenital heart disease [37,38].

Different exercise protocols have been tested, assessing the efficacy and safety of mixed aerobic and resistance exercise after appropriate counseling and risk stratification [7,9,39,40,41,42,43,44,45]

More recently, high-intensity interval training has been effective in improving VO2 max in patients with coronary artery disease [46,47] but was not superior compared to moderate continuous training in patients with heart failure during cardiac rehabilitation [48,49].

However, previous studies have shown that several factors may promote a more sedentary lifestyle, including cardiac and respiratory limitations [13,50], inappropriate advice regarding exercise [51], and overprotection by the family environment [52].

In this setting, the recent COVID-19 pandemic has dramatically changed not only working and life habits [22,53,54,55] but also access to medical care, promoting remote consultations [56,57].

Many studies have established the feasibility, safety, and efficacy of personalized home PA prescription [9,58,59], with home exercise protocols aiming to achieve targeted HR based on exercise performance [9,17].

However, patients with complex anatomy, hemodynamically significant residual lesions, or high-risk ECG features require closer supervision [39,60,61]. In this context, recent technological advancements, such as physical activity trackers and applications for smartwatches and smartphones that monitor vital parameters (heart rate, blood pressure, and oxygen saturation), have shown to be sensitive and specific in detecting irregular heart rate [62] and provide single or multiple lead ECGs, may significantly aid in both remote medical consults and in supervised exercise sessions, aiming to improve or at least maintain stable the degree of PA in adult patients with congenital heart disease.

### 4.3. Limitation of the Study

Our study has some limitations. First, the number of patients studied is comparable to that of other similar studies [63,64]. Furthermore, all our patients were investigated retrospectively. Although our population encompassed ACHD patients, it was heterogeneous, as both simple and complex congenital heart disease were included. Finally, VO2 max was available in 78.3% of patients.

## 5. Conclusions

Our study highlights the pivotal role of exercise in ACHD patients and the detrimental consequences that physical inactivity can produce in the short term, reducing exercise duration and VO2 max. During periods of restricted mobility, tailored exercise routines and supervised virtual exercise programs, where indicated, could help ensure continuity of exercise in ACHD patients and improve exercise self-confidence. Healthcare providers could encourage an active lifestyle and regular PA in adults with congenital heart disease unless contraindicated.

## Figures and Tables

**Table 1:** Baseline characteristics of 37 ACHD patients.

		Tot (37)	Group A (17)	Group B (20)
Age (years)		26 (6)	26 (6)	26 (7)
Gender (Male)		22 (55.6)	12 (71)	10 (50)
CHD	rTOF	15 (40.5)	7 (41)	8 (40)
	Fontan	8 (21.6)	3 (18)	5 (25)
	rCoA	5 (13.5)	2 (12)	3 (15)
	Valvular	4 (10.8)	3 (18)	1 (5)
	TGA Arterial switch	2 (5.4)	1 (6)	1 (5)
	AVSD	1 (2.7)	1 (6)	-
	PA-VSD	1 (2.7)	-	1 (5)
	TGA Rastelli Repair	1 (2.7)	-	1 (5)
NYHA	I	30 (81.1)	16 (94)	14 (70)
	II	7 (18.4)	1 (6)	6 (30)

rTOF repaired tetralogy of Fallot, rCoA repaired coarctation of the aorta, TGA transposition of the great arteries, AVSD atrioventricular septal defect, and PA-VSD pulmonary atresia with ventricular septal defect.

**Table 2:** Characteristics of the two groups at baseline (pre-LD) and after one year (post-LD).

	Group A			Group B		
	Pre-LD	Post-LD	*p*-Value	Pre-LD	Post-LD	*p*-Value
RPA min/week, mean (SD)	115 (46)	91 (64)	0.03	-	-	-
Weight (kg), median (range)	63 (51–100)	68 (49–98)	0.092	62.5 (47–84)	64 (45–85)	0.067
BMI, mean (SD)	22.0 (2.7)	22.6 (2.9)	0.130	22.8 (3)	23.0 (3.4)	0.318
Duration (s), median (range)	625 (546–900)	581 (350–720)	0.009	630 (383–750)	578 (321–720)	0.118
HR max (%), median (range)	90 (76–97)	87 (70–95)	0.674	86 (69–93)	84 (56–97)	0.496
VO2max (mL/kg/min), mean (SD)	31.3 (5.5)	28.0 (4.1)	0.015	24.9 (4.7)	25.1 (5.5)	0.820

RPA: regular physical activity, BMI: body-mass index, HR max: achieved percentage heart rate, VO2 max: maximal oxygen consumption, LD: lockdown. Normally distributed variables are presented as mean (SD) and differences pre- and post-LD within the same group assessed with paired Student *t*-tests. Skewed variables are presented as median (range), and differences pre- and post-LD within the same group are assessed with the Wilcoxon test.

## Data Availability

The data presented in this study are available on request from the corresponding author.

## References

[1] Buckley B.J.R., Harrison S.L., Fazio-Eynullayeva E., Underhill P., Jones I.D., Williams N., Lip G.Y.H. (2022). Exercise rehabilitation associates with lower mortality and hospitalisation in cardiovascular disease patients with COVID-19. Eur. J. Prev. Cardiol..

[2] Zaccagni L., Toselli S., Barbieri D. (2021). Physical Activity during COVID-19 Lockdown in Italy: A Systematic Review. Int. J. Environ. Res. Public Health.

[3] Franco I., Bianco A., Bonfiglio C., Sorino P., Mirizzi A., Campanella A., Buongiorno C., Liuzzi R., Osella A.R. (2021). Decreased levels of physical activity: Results from a cross-sectional study in southern Italy during the COVID-19 lockdown. J. Sport. Med. Phys. Fitness.

[4] Sepúlveda-Loyola W., Rodríguez-Sánchez I., Pérez-Rodríguez P., Ganz F., Torralba R., Oliveira D.V., Rodríguez-Mañas L. (2020). Impact of Social Isolation Due to COVID-19 on Health in Older People: Mental and Physical Effects and Recommendations. J. Nutr. Health Aging.

[5] Wunsch K., Kienberger K., Niessner C. (2022). Changes in Physical Activity Patterns Due to the COVID-19 Pandemic: A Systematic Review and Meta-Analysis. Int. J. Environ. Res. Public Health.

[6] Valenzuela P.L., Ruilope L.M., Santos-Lozano A., Wilhelm M., Kränkel N., Fiuza-Luces C., Lucia A. (2023). Exercise benefits in cardiovascular diseases: From mechanisms to clinical implementation. Eur. Heart J..

[7] Tran D., Maiorana A., Ayer J., Lubans D.R., Davis G.M., Celermajer D.S., d’Udekem Y., Cordina R. (2020). Recommendations for exercise in adolescents and adults with congenital heart disease. Prog. Cardiovasc. Dis..

[8] Pelliccia A., Fagard R., Bjørnstad H.H., Anastassakis A., Arbustini E., Assanelli D., Biffi A., Borjesson M., Carrè F., Corrado D. (2005). Recommendations for competitive sports participation in athletes with cardiovascular disease: A consensus document from the Study Group of Sports Cardiology of the Working Group of Cardiac Rehabilitation and Exercise Physiology and the Working Group of Myocardial and Pericardial Diseases of the European Society of Cardiology. Eur. Heart J..

[9] Perrone M.A., Pomiato E., Palmieri R., Di Già G., Piemonte F., Porzio O., Gagliardi M.G. (2022). The Effects of Exercise Training on Cardiopulmonary Exercise Testing and Cardiac Biomarkers in Adult Patients with Hypoplastic Left Heart Syndrome and Fontan Circulation. J. Cardiovasc. Dev. Dis..

[10] Bredy C., Ministeri M., Kempny A., Alonso-Gonzalez R., Swan L., Uebing A., Diller G.P., Gatzoulis M.A., Dimopoulos K. (2018). New York Heart Association (NYHA) classification in adults with congenital heart disease: Relation to objective measures of exercise and outcome. Eur. Heart J. Qual. Care Clin. Outcomes.

[11] Diller G.P., Kempny A., Alonso-Gonzalez R., Swan L., Uebing A., Li W., Babu-Narayan S., Wort S.J., Dimopoulos K., Gatzoulis M.A. (2015). Survival Prospects and Circumstances of Death in Contemporary Adult Congenital Heart Disease Patients Under Follow-Up at a Large Tertiary Centre. Circulation.

[12] Kempny A., Dimopoulos K., Uebing A., Moceri P., Swan L., Gatzoulis M.A., Diller G.P. (2012). Reference values for exercise limitations among adults with congenital heart disease. Relation to activities of daily life--single centre experience and review of published data. Eur. Heart J..

[13] Dimopoulos K., Diller G.P., Piepoli M.F., Gatzoulis M.A. (2006). Exercise intolerance in adults with congenital heart disease. Cardiol. Clin..

[14] Müller J., Hager A., Diller G.P., Derrick G., Buys R., Dubowy K.O., Takken T., Orwat S., Inuzuka R., Vanhees L. (2015). Peak oxygen uptake, ventilatory efficiency and QRS-duration predict event free survival in patients late after surgical repair of tetralogy of Fallot. Int. J. Cardiol..

[15] Giardini A., Hager A., Lammers A.E., Derrick G., Müller J., Diller G.P., Dimopoulos K., Odendaal D., Gargiulo G., Picchio F.M. (2009). Ventilatory efficiency and aerobic capacity predict event-free survival in adults with atrial repair for complete transposition of the great arteries. J. Am. Coll. Cardiol..

[16] Inuzuka R., Diller G.P., Borgia F., Benson L., Tay E.L., Alonso-Gonzalez R., Silva M., Charalambides M., Swan L., Dimopoulos K. (2012). Comprehensive use of cardiopulmonary exercise testing identifies adults with congenital heart disease at increased mortality risk in the medium term. Circulation.

[17] Gentili F., Cafiero G., Perrone M.A., Bianco M., Salvati A., Giordano U., Silva Kikina S., Guccione P., De Zorzi A., Galletti L. (2021). The Effects of Physical Inactivity and Exercise at Home in Young Patients with Congenital Heart Disease during the COVID-19 Pandemic. Int. J. Environ. Res. Public Health.

[18] Delisle Nyström C., Alexandrou C., Henström M., Nilsson E., Okely A.D., Wehbe El Masri S., Löf M. (2020). International Study of Movement Behaviors in the Early Years (SUNRISE): Results from SUNRISE Sweden’s Pilot and COVID-19 Study. Int. J. Environ. Res. Public Health.

[19] Bronikowska M., Krzysztoszek J., Łopatka M., Ludwiczak M., Pluta B. (2021). Comparison of Physical Activity Levels in Youths before and during a Pandemic Lockdown. Int. J. Environ. Res. Public Health.

[20] Cheval B., Sivaramakrishnan H., Maltagliati S., Fessler L., Forestier C., Sarrazin P., Orsholits D., Chalabaev A., Sander D., Ntoumanis N. (2021). Relationships between changes in self-reported physical activity, sedentary behaviour and health during the coronavirus (COVID-19) pandemic in France and Switzerland. J. Sport. Sci..

[21] Srivastav A.K., Sharma N., Samuel A.J. (2021). Impact of Coronavirus disease-19 (COVID-19) lockdown on physical activity and energy expenditure among physiotherapy professionals and students using web-based open E-survey sent through WhatsApp, Facebook and Instagram messengers. Clin. Epidemiol. Glob. Health.

[22] Karuc J., Sorić M., Radman I., Mišigoj-Duraković M. (2020). Moderators of Change in Physical Activity Levels during Restrictions Due to COVID-19 Pandemic in Young Urban Adults. Sustainability.

[23] Radke R.M., Frenzel T., Baumgartner H., Diller G.P. (2020). Adult congenital heart disease and the COVID-19 pandemic. Heart.

[24] Diller G.P., Gatzoulis M.A., Broberg C.S., Aboulhosn J., Brida M., Schwerzmann M., Chessa M., Kovacs A.H., Roos-Hesselink J. (2021). Coronavirus disease 2019 in adults with congenital heart disease: A position paper from the ESC working group of adult congenital heart disease, and the International Society for Adult Congenital Heart Disease. Eur. Heart J..

[25] Koletzko B., Holzapfel C., Schneider U., Hauner H. (2021). Lifestyle and Body Weight Consequences of the COVID-19 Pandemic in Children: Increasing Disparity. Ann. Nutr. Metab..

[26] Kreutz R., Dobrowolski P., Prejbisz A., Algharably E.A.E., Bilo G., Creutzig F., Grassi G., Kotsis V., Lovic D., Lurbe E. (2021). Lifestyle, psychological, socioeconomic and environmental factors and their impact on hypertension during the coronavirus disease 2019 pandemic. J. Hypertens..

[27] Brida M., Gatzoulis M.A. (2019). Adult congenital heart disease: Past, present and future. Acta Paediatr..

[28] Clark A.L., Gatzoulis M.A., Redington A.N. (1995). Ventilatory responses to exercise in adults after repair of tetralogy of Fallot. Br. Heart J..

[29] Diller G.P., Giardini A., Dimopoulos K., Gargiulo G., Müller J., Derrick G., Giannakoulas G., Khambadkone S., Lammers A.E., Picchio F.M. (2010). Predictors of morbidity and mortality in contemporary Fontan patients: Results from a multicenter study including cardiopulmonary exercise testing in 321 patients. Eur. Heart J..

[30] Diller G.P., Dimopoulos K., Okonko D., Uebing A., Broberg C.S., Babu-Narayan S., Bayne S., Poole-Wilson P.A., Sutton R., Francis D.P. (2006). Heart rate response during exercise predicts survival in adults with congenital heart disease. J. Am. Coll. Cardiol..

[31] Diller G.P., Dimopoulos K., Okonko D., Li W., Babu-Narayan S.V., Broberg C.S., Johansson B., Bouzas B., Mullen M.J., Poole-Wilson P.A. (2005). Exercise intolerance in adult congenital heart disease: Comparative severity, correlates, and prognostic implication. Circulation.

[32] Leonardi B., Gentili F., Perrone M.A., Sollazzo F., Cocomello L., Silva Kikina S., Wald R.M., Palmieri V., Secinaro A., Gagliardi M.G. (2022). Cardiopulmonary Exercise Testing in Repaired Tetralogy of Fallot: Multiparametric Overview and Correlation with Cardiac Magnetic Resonance and Physical Activity Level. J. Cardiovasc. Dev. Dis..

[33] Lin K.L., Pan J.Y., Chen G.B., Liou I.H., Weng K.P., Li C.H., Tuan S.H. (2021). Serial Cardiopulmonary Exercise Testing in Patients after Extracardiac Conduit Total Cavopulmonary Connection for Single-Ventricle Hearts: An Observational Study. J. Pediatr..

[34] Illinger V., Materna O., Slabý K., Jičínská D., Kovanda J., Koubský K., Pokorný J., Procházka M., Antonová P., Hoskovec A. (2022). Exercise capacity after total cavopulmonary anastomosis: A longitudinal paediatric and adult study. ESC Heart Fail..

[35] Baumgartner H., De Backer J., Babu-Narayan S.V., Budts W., Chessa M., Diller G.P., Lung B., Kluin J., Lang I.M., Meijboom F. (2021). 2020 ESC Guidelines for the management of adult congenital heart disease. Eur. Heart J..

[36] Perrone M.A., Iellamo F., D’Antoni V., Gismondi A., Di Biasio D., Vadalà S., Marazzi G., Morsella V., Volterrani M., Caminiti G. (2023). Acute Changes on Left Atrial Function during Incremental Exercise in Patients with Heart Failure with Mildly Reduced Ejection Fraction: A Case-Control Study. J. Pers. Med..

[37] Bay A., Sandberg C., Thilén U., Wadell K., Johansson B. (2018). Exercise self-efficacy in adults with congenital heart disease. Int. J. Cardiol. Heart Vasc..

[38] Chaix M.A., Marcotte F., Dore A., Mongeon F.P., Mondésert B., Mercier L.A., Khairy P. (2016). Risks and Benefits of Exercise Training in Adults with Congenital Heart Disease. Can. J. Cardiol..

[39] Anderson C.A.J., Suna J.M., Keating S.E., Cordina R.M.B.B.S., Tran D.L., Ayer J.M.B.B.S., Coombes J.S. (2022). Safety and efficacy of exercise training in children and adolescents with congenital heart disease: A systematic review and descriptive analysis. Am. Heart J..

[40] Cordina R.L., O’Meagher S., Karmali A., Rae C.L., Liess C., Kemp G.J., Puranik R., Singh N., Celermajer D.S. (2013). Resistance training improves cardiac output, exercise capacity and tolerance to positive airway pressure in Fontan physiology. Int. J. Cardiol..

[41] Shiraz S., Salimei C., Aracri M., Di Lorenzo C., Farsetti P., Parisi A., Iellamo F., Caminiti G., Perrone M.A. (2024). The Effects of High-Intensity Interval Training on Cognitive and Physical Skills in Basketball and Soccer Players. J. Funct. Morphol. Kinesiol..

[42] Caminiti G., Perrone M.A., Volterrani M., Iellamo F., Marazzi G., Selli S., Franchini A., Padua E. (2022). Effects of Concurrent Aerobic Plus Resistance Training on Blood Pressure Variability and Blood Pressure Values in Patients with Hypertension and Coronary Artery Disease: Gender-Related Differences. J. Cardiovasc. Dev. Dis..

[43] Caminiti G., Perrone M.A., Iellamo F., D’Antoni V., Catena M., Franchini A., Volterrani M. (2022). Acute Left Atrial Response to Different Eccentric Resistance Exercise Loads in Patients with Heart Failure with Middle Range Ejection Fraction: A Pilot Study. J. Pers. Med..

[44] Volterrani M., Caminiti G., Perrone M.A., Cerrito A., Franchini A., Manzi V., Iellamo F. (2023). Effects of Concurrent, Within-Session, Aerobic and Resistance Exercise Training on Functional Capacity and Muscle Performance in Elderly Male Patients with Chronic Heart Failure. J. Clin. Med..

[45] Caminiti G., Volterrani M., Iellamo F., Marazzi G., Manzi V., D’Antoni V., Vadalà S., Di Biasio D., Catena M., Morsella V. (2024). Changes in left atrial function following two regimens of combined exercise training in patients with ischemic cardiomyopathy: A pilot study. Front. Cardiovasc. Med..

[46] Yu H., Zhao X., Wu X., Yang J., Wang J., Hou L. (2023). High-intensity interval training versus moderate-intensity continuous training on patient quality of life in cardiovascular disease: A systematic review and meta-analysis. Sci. Rep..

[47] Wang C., Xing J., Zhao B., Wang Y., Zhang L., Wang Y., Zheng M., Liu G. (2022). The Effects of High-Intensity Interval Training on Exercise Capacity and Prognosis in Heart Failure and Coronary Artery Disease: A Systematic Review and Meta-Analysis. Cardiovasc. Ther..

[48] Mueller S., Winzer E.B., Duvinage A., Gevaert A.B., Edelmann F., Haller B., Pieske-Kraigher E., Beckers P., Bobenko A., Hommel J. (2021). Effect of High-Intensity Interval Training, Moderate Continuous Training, or Guideline-Based Physical Activity Advice on Peak Oxygen Consumption in Patients with Heart Failure with Preserved Ejection Fraction: A Randomized Clinical Trial. JAMA.

[49] Ellingsen Ø., Halle M., Conraads V., Støylen A., Dalen H., Delagardelle C., Larsen A.I., Hole T., Mezzani A., Van Craenenbroeck E.M. (2017). High-Intensity Interval Training in Patients with Heart Failure with Reduced Ejection Fraction. Circulation.

[50] Bassareo P.P., Saba L., Solla P., Barbanti C., Marras A.R., Mercuro G. (2014). Factors influencing adaptation and performance at physical exercise in complex congenital heart diseases after surgical repair. BioMed Res. Int..

[51] Swan L., Hillis W.S. (2000). Exercise prescription in adults with congenital heart disease: A long way to go. Heart.

[52] Reybrouck T., Mertens L. (2005). Physical performance and physical activity in grown-up congenital heart disease. Eur. J. Cardiovasc. Prev. Rehabil..

[53] Muscogiuri G., Pugliese G., Barrea L., Savastano S., Colao A. (2020). Commentary: Obesity: The “Achilles heel” for COVID-19?. Metabolism.

[54] O’Neill C.D., Vidal-Almela S., Tulloch H.E., Coutinho T., Prince S.A., Reed J.L. (2021). COVID-19 pandemic—Inequities and inequalities to exercise and their consequences on the physical and mental health of women with cardiovascular disease: Recommendations on how to address the needs of women. Appl. Physiol. Nutr. Metab..

[55] Umutlu G., Acar N.E., Sinar D.S., Akarsu G., Güven E., Yildirim I. (2022). COVID-19 and physical activity in sedentary individuals: Differences in metabolic, cardiovascular, and respiratory responses during aerobic exercise performed with and without a surgical face masks. J. Sport. Med. Phys. Fitness.

[56] Anthony J., Prabhakar C.R.K., Clift P., Hudsmith L. (2021). COVID-19 and adult congenital heart disease services: Impact on support and advice from nurse specialists. Br. J. Nurs..

[57] Carrillo de Albornoz S., Sia K.L., Harris A. (2022). The effectiveness of teleconsultations in primary care: Systematic review. Fam. Pract..

[58] Pietrobelli A., Pecoraro L., Ferruzzi A., Heo M., Faith M., Zoller T., Antoniazzi F., Piacentini G., Fearnbach S.N., Heymsfield S.B. (2020). Effects of COVID-19 Lockdown on Lifestyle Behaviors in Children with Obesity Living in Verona, Italy: A Longitudinal Study. Obesity.

[59] Bentlage E., Ammar A., How D., Ahmed M., Trabelsi K., Chtourou H., Brach M. (2020). Practical Recommendations for Maintaining Active Lifestyle during the COVID-19 Pandemic: A Systematic Literature Review. Int. J. Environ. Res. Public Health.

[60] Khoury M., Cordina R. (2022). Exercise Training for People Living with Fontan Circulation: An Underutilized Intervention. Can. J. Cardiol..

[61] Wood G., Scheer A., Saundankar J., Tran D., Cordina R., Maiorana A. (2024). The effects of telerehabilitation in adults with complex biventricular congenital heart conditions: Protocol for a multi-centre, randomised controlled trial-CH-FIT. Trials.

[62] Hindricks G., Potpara T., Dagres N., Arbelo E., Bax J.J., Blomström-Lundqvist C., Boriani G., Castella M., Dan G.A., Dilaveris P.E. (2021). 2020 ESC Guidelines for the diagnosis and management of atrial fibrillation developed in collaboration with the European Association for Cardio-Thoracic Surgery (EACTS): The Task Force for the diagnosis and management of atrial fibrillation of the European Society of Cardiology (ESC) Developed with the special contribution of the European Heart Rhythm Association (EHRA) of the ESC. Eur. Heart J..

[63] Powell A.W., Mays W.A., Wittekind S.G., Chin C., Knecht S.K., Lang S.M., Opotowsky A.R. (2023). Stable fitness during COVID-19: Results of serial testing in a cohort of youth with heart disease. Front. Pediatr..

[64] Aronoff E.B., Chin C., Opotowsky A.R., Mays W.A., Knecht S.K., Goessling J., Rice M., Shertzer J., Wittekind S.G., Powell A.W. (2024). Facility-Based and Virtual Cardiac Rehabilitation in Young Patients with Heart Disease During the COVID-19 Era. Pediatr. Cardiol..

